# Evidence of Pulmonary Hypertension after SARS-CoV-2 Infection in Subjects without Previous Significant Cardiovascular Pathology

**DOI:** 10.3390/jcm10020199

**Published:** 2021-01-07

**Authors:** Cristina Tudoran, Mariana Tudoran, Voichita Elena Lazureanu, Adelina Raluca Marinescu, Gheorghe Nicusor Pop, Alexandru Silvius Pescariu, Alexandra Enache, Talida Georgiana Cut

**Affiliations:** 1 Department VII, Internal Medicine II, Discipline of Cardiology, University of Medicine and Pharmacy “Victor Babes” Timisoara, E. Murgu Square, Nr. 2, 300041 Timisoara, Romania; tudoran.cristina@umft.ro; 2 Department XIII, Discipline of Infectious Diseases, University of Medicine and Pharmacy “Victor Babes” Timisoara, E. Murgu Square, Nr. 2, 300041 Timisoara, Romania; vlazureanu@gmail.com (V.E.L.); marinescu.adelina@umft.ro (A.R.M.); 3 University of Medicine and Pharmacy “Victor Babes” Timisoara, E. Murgu Square, Nr. 2, 300041 Timisoara, Romania; pop.nicusor@umft.ro (G.N.P.); alex.pescariu@yahoo.com (A.S.P.); Talida.cut@gmail.com (T.G.C.); 4 Department VIII, Discipline of Forensic Medicine, University of Medicine and Pharmacy “Victor Babes” Timisoara, E. Murgu Square, Nr. 2, 300041 Timisoara, Romania; enache.alexandra@umft.ro

**Keywords:** COVID-19, thorax CT COVID score, pulmonary hypertension, estimates systolic pulmonary pressure, right ventricle global longitudinal strain

## Abstract

Background: Coronavirus (Covid-19) infection represents a serious medical condition, often associated with cardiovascular complications, pulmonary hypertension (PH), and right ventricle dysfunction (RVD). The aim of this study is to show, by means of transthoracic echocardiography (TTE), the presence of an increased estimated systolic pressure in the pulmonary artery (esPAP) and altered right ventricular global longitudinal strain (RV-GLS) in patients without history of PH. Methods: In a group of 91 patients, aged under 55 years, hospitalized for a moderate Covid-19 infection, a thorough cardiologic and TTE examination were performed two months after discharge. Their initial thorax computer-tomography (TCT) images and laboratory data were accessed from the electronic data base of the hospital. Results: We observed an increased prevalence of PH (7.69%) and RVD (10.28%), significantly correlated with the initial levels of the TCT score and inflammatory factors (*p* ˂ 0.001), but borderline changes were observed in more patients. Multivariate regression analysis showed that these factors and RV-GLS explain 89.5% of elevated esPAP. Conclusions: In COVID-19 infection, PH and RVD are common complications, being encountered after the recovery even in moderate cases. It appears to be a connection between their severity and the extent of the initial pulmonary injury and of the inflammatory response.

## 1. Introduction

Coronavirus pandemic infection (COVID-19), determined by the coronavirus 2 (SARS-CoV-2), represents a serious medical condition, characterized mainly by severe pneumonia, which is often associated with acute respiratory distress syndrome (ARDS), respiratory failure (RF), and multiple organ dysfunction/failure [1,2]. Cardiovascular complications are frequent (20–30% of cases) and represent an important negative prognostic factor [3,4]. Pulmonary involvement is bilateral, consisting of extensive interstitial and alveolar inflammatory infiltrates, thickening of alveolar septa, vascular congestion, and lung edema, which could be responsible, in some patients, for the development of pulmonary fibrosis. As a consequence of lung parenchymal damage and of altered pulmonary circulation, pulmonary hypertension (PH) may develop, leading to right ventricular (RV) involvement and right heart failure (RHF) [5]. The pathophysiology of this type of PH is complex and multifactorial and mechanisms such as oxidative stress, mitochondrial dysfunction, and DNA damage, inflammation, hypoxia, associated with endothelial dysfunction, and pulmonary micro-embolism have been considered potential factors for the alterations of pulmonary circulation [6]. It has been proposed that this type of PH should be considered a combination between PH of group 3 (due to fibrosis and/or obstructive lung disease) and 4 (due to pulmonary artery obstructions) [7], but the contribution of an immunological response that could explain the perpetuation of PH was not ruled out. In recent studies, the suggested prevalence of PH in patients with COVID-19 is around 13%, but its further evolution remains unclear [5]. Unfortunately, due to qualified personal shortage and to avoid contamination, a thorough TTE is seldom performed, especially in patients with moderate/mild COVID-19 forms and the diagnosis of PH could be missed in this early phase, emerging later as an unexplained finding.

The severity of PH can be evaluated quite accurately noninvasively, by means of transthoracic echocardiography (TTE), which allows the assessment of parameters characterizing the RV dimension and function and the estimation of the systolic pressure in the pulmonary artery (esPAP) [8]. Similarly, the extent of the pulmonary damage in the acute phase of a COVID-19 infection, can be determined adequately by thorax computer tomography (TCT). Several methods are being proposed for the quantitative computation of pulmonary lesions [9,10], but their prognostic value remains a matter of debate [11].

The aim of this study is to illustrate the presence and severity of PH at two months after the recovery from a mild/moderate COVID-19 pulmonary infection in subjects without previous significant cardiovascular or pulmonary diseases that could explain the existence of PH. Another aim was to show if the severity of PH is correlated with the extent of the initial lung involvement, assessed by means of an initial radiological COVID score, and the alterations of inflammatory markers.

## 2. Experimental Section

### 2.1. Study Population

By analyzing the electronic medical data base, of all 557 patients hospitalized during 15 April and 15 July 2020 for COVID-19 in the two clinics of the Hospital for Infectious Diseases from Timisoara, we observed that 507 patients were discharged alive after a median of 9.7 (8.5 to 10) days of hospitalization. Of the 350 patients with mild/moderate forms of pneumonia according to the WHO’s criteria [12], 153 subjects, not registered with significantly associated cardiovascular pathology, were considered suitable for our study. After two months from discharge, they were contacted by phone to attend a cardiologic exam and a transthoracic echocardiography. Furthermore, 117 patients from our county agreed to take part in the study. All patients were carefully evaluated by a cardiologist in order to identify any pathological cardiovascular findings that could explain the presence of PH and 26 subjects were excluded due to associated medical conditions. For the remaining 91 participants, their baseline clinical characteristics, radiological and laboratory data, as well as their therapy during hospitalization were obtained, retrospectively, by reviewing their electronic medical records. The Local Scientific Research Ethics Committee of our hospital approved the design and methodology of our study (No. 20715/17.10.2020). Before data collection, all patients included in this evaluation had signed an individual informed consent form.

#### 2.1.1. Inclusion Criteria

The inclusion criteria included subjects with a recent hospitalization for COVID-19, confirmed by a positive result of real-time reverse transcriptase–polymerase chain reaction (RT-PCR) assay of nasal and pharyngeal swabs, two months ago, with mild/moderate pneumonia, without respiratory insufficiency needing mechanical ventilation, and without previous cardiovascular diseases.

#### 2.1.2. Exclusion Criteria

The exclusion criteria involve patients with severe/critical form of COVID-19 pulmonary infection, those without a thorax CT exam during hospitalization, patients diagnosed with idiopathic PH or other forms belonging to group 1, patients with significant cardiovascular pathology leading to PH of group 2, and the ones with respiratory diseases, causing group 3 PH, as well as those with a history of pulmonary thromboembolism that could cause PH of group 4. We also excluded patients with metabolic, systemic, hematological, or other pathology that could lead to a PH of group 5, as well as those treated with vasodilators used in the therapy of PH (prostacyclin analogues or endothelin receptor antagonists, phosphodiesterase type 5 inhibitors, and guanylate cyclase stimulators).

### 2.2. Methods

#### 2.2.1. TCT

Although all patients had a TCT cross evaluation of the lung injury (under 40% of the lung parenchyma) during admission. In order to increase the accuracy of our results, a semi-quantitative TCT re-assessment of pulmonary lesions, according to the severity scoring proposed by Pan et al. [13], was performed at the beginning of the study. A global TCT COVID score (0 to 25) was calculated as the sum of the scores for each of the five lobes, taking into account the extent of the lung parenchyma damage (0: no injury, 1: <5% injury, 2: 5–25% lesions, 3: 26–50% involvement, 4: 51–75% injury and 5: >75% involvement), with higher values indicating a greater alteration of lung parenchyma.

#### 2.2.2. Echocardiographic Examination

For the purposes of avoiding inter-observer differences, the same experienced operator performed all TTE assessments, according to guidelines (14). After a regular exam of the cardiac morphology and function, we assessed the following parameters.

1. Right atrial (RA) diameter, in a four-chamber view.
2. Right ventricle (RV) diameter, measured in a four-chamber view, under the tricuspid annulus.
3. Tricuspid annular plane systolic excursion (TAPSE), values under 17 mm, measured at the lateral tricuspid valve annulus in M-Mode, were considered suggestive for RV dysfunction.
4. Tricuspid Regurgitation Velocity (TRV), determined in a continuous Doppler.
5. Echo-cardiographically estimated systolic PAP (esPAP), based on the peak TRV, taking into account the right atrial pressure (RAP), determined by assessing the inferior vena cava diameter, as well as its respiratory variations. In this study, we considered that esPAP values of ≥35 mm Hg at rest, indicates PH [3,10] with the severity ranging from mild (35–44 mm Hg), to moderate (45–60 mm Hg) and severe (>60 mm Hg) [14,15].
6. Pulmonary Vascular Resistance (PVR) was estimated by reporting the peak TRV (m/sec) to the RV outflow tract velocity-time integral (cm), allowing an acceptable noninvasive evaluation. In this study, we considered PVR of >2 WU as elevated and PVR of >3 WU as significant [16,17].
7. Right ventricular global longitudinal strain (RV-GLS) was performed in an apical four chamber view [18]. After tracing the RV endocardial border, the region of interest was automatically generated, and manual corrections were subsequently performed to fit the thickness of the RV myocardial wall [19]. According to the latest international recommendations, RV dysfunction (RVD) was defined as either TAPSE under <17 mm and/or RV-GLS under −28% (borderline values for TAPSE were 17–20 and for RV-GLS −25 to −27) [17,18].

### 2.3. Statistical Analysis 

Data analysis was performed using the Statistical Package for the Social Sciences v.25 (SPSS, Chicago, IL, USA). Continuous variables were presented as mean and standard deviation (SD) or median and interquartile range (IQR), and categorical variables were presented as frequency and percentages. The results of the normality test (Shapiro-Wilk) showed a non-Gaussian distribution, suggesting that we should continue the analysis by using nonparametric tests. For the evaluation of the potential connection between esPAP, RV-GLS, and laboratory results, we employed Spearman’s correlation test. The individual impact of several confounding factors on the variance of continuous variables was assessed by building multivariate regression models. The quality of the model was described by using the accuracy of prediction and R squared. The predictors, in the final regression equations, were accepted according to a repeated backward-stepwise algorithm (inclusion criteria *p* < 0.05, exclusion criteria *p* > 0.10) in order to obtain the most appropriate theoretical model to fit the collected data. We considered a *p* value < 0.05 to indicate a statistically significant difference.

## 3. Results

We included in our study 91 patients, aged between 30 and 55 years, median age 46 (40–50) years, hospitalized for a mild/moderate COVID-19 pulmonary infection, but without severe respiratory failure needing mechanical ventilation. Forty-seven of them were women and forty-four men, 30.76% of them were overweight and 25.27% obese. Their TCTs, performed during hospitalization, were re-examined. The TCT COVID score was calculated and their laboratory data were obtained, retrospectively, from the electronic hospital records (Table 1). The majority of these patients had a basic normal TTE, performed mainly in the emergency room or during the hospitalization, with an abbreviated assessment of cardiac dimensions and function. This evaluation did not include accurate measurements of all studied parameters, but no cardiac abnormalities were mentioned. All patients were treated with protease inhibitors (remdesivir (Gilead Sciences, Foster City, CA, USA), lopinavir/ritonavir (AbbVie, Ludwigshafen, Germany)), antibiotics (cefuroxime (Antibiotice, Iasi, Romania) or azithromycine (Sandoz, Targu-Mures, Romania)), and anticoagulants (apixaban (Bristol-Myers Squibb/Phizer EEIG, Dublin, Ireland) or rivaroxaban (Bayer AG, Leverkusen, Germany)) in a prophylactic dosage, continued 40 days after discharge.

At two months after discharge, when the patients included in our study group should have been totally recovered, they underwent a thorough clinical and TTE exam in order to identify the presence of PH or/and of RV dysfunction that could have developed during COVID-19, persisting even after the recovery phase. These data are presented in Table 1. The assessment of esPAP and the detailed examination of RV function, evidenced in seven subjects (7.69%), the presence of PH (all with esPAP ˃ 35 mmHg, six of them with PVR ˃ 3 WU) and, in 10 cases (10.28%), the existence of RVD, with six of them having both dysfunctions. We observed in the other 11 patients (19.789%), borderline values of esPAP (over 30, but under 35 mmHg) and PVR (between 2 and 3 WU), which is higher than expected in their category of age, and 12 (13.18%) had associated borderline levels of RV-GLS and of TAPSE when taking into account that, in our study group, younger patients (under 55 years) were included.

Deliberating over possible connections between these TTE findings and TCT aspects and/or biological markers of inflammation, we performed a statistical analysis and showed statistically significant moderate correlations between esPAP values and TCT Covid scores as well as with CRP and interleukin-6 levels and with the number of days until the negativation of PCR (Table 2). The highest correlations were observed between esPAP levels and the inflammatory factors. Similar results were obtained for RV-GLS (Table 2).

As we analyzed the relations between esPAP, PVR, and the parameters characterizing RV function, we detected moderate, but statistically significant correlation (*p* < 0.001) between esPAP and PVR (*r* = 0.686, 95%CI (0.525; 0.802)), RV-GLS (*r* = 0.656, 95%CI (0.479; 0.799)), and TAPSE (*r* = −0.644, 95%CI (−0.792; −0.465)) and between RV-GLS and TAPSE (*r* = −0.644, 95%CI (−0.792; −0.465), *p* < 0.001).

Multivariate linear regression analysis was used to evaluate the independent predictor factors for the development of PH and RV-GLS in our patients’ group. The regression model was built based on the forward stepwise method, and Akaike information criteria (AIC) were used to appreciate the best model (Table 3). After adjustment for gender, age, and BMI as potential confounding factors, we identified the most significant predictor factors for the occurrence of PH and RVD in our patient group (Table 3). Our results highlighted that the initial values of some laboratory data like global TCT COVID score, D-dimers, fibrinogen, interleukin-6, and O_2_ saturation, determined during hospitalization, as well as echocardiographic parameters like PVR, assessed after recovery, explain 88.5% of the elevated levels of esPAP (R2 = 0.885), while the time until the normalization of PCR was not significant (Table 3). A similar impact of the initial TCT COVID-score and of inflammatory factors, determined during hospitalization, and of several echocardiographic parameters assessed after recovery (TRVmax, esPAP, RA diameter) on the RV-GLS levels were also observed (R2 = 0.875).

## 4. Discussion

The increased prevalence of cardiovascular complications in COVID-19 is a debated topic in recent studies [1,3,12,20,21]. Especially PH and RVD were frequently documented, mostly in critically ill patients, with acute respiratory distress syndrome and respiratory failure [5,6,12], treated in Intensive Care Units. The incidence of PH has been appreciated around 15% [5], but it could be much higher when taking into account the difficulty of performing TTE in severely ill, often mechanically ventilated, patients [22,23]. Actual studies analyzed patients with COVID-19 altogether, with most of them being older people with comorbidities, mainly associated cardiovascular diseases [24]. Another aspect is that many published articles present data on severe or critically cases, with less concern on patients with mild or moderate forms, which represent 80% of all infections. Considering this aspect, a real prevalence of PH, due only to COVID-19, is very difficult to assess. This is the mean reason why, in this study, we focused only on patients under 55 years old, who suffered with a moderate form of COVID-19 pneumonia, without respiratory failure requiring mechanical ventilation. In order to estimate the prevalence of PH due only to this pulmonary infection, we selected in our study only subjects without known pathology that could explain the presence of PH.

PH can develop progressively, but cases with rapid progress were also described [7]. The evolution of this type of PH has not yet been established. It is assumed that it alleviates gradually, during recovery from COVID-19, but its real evolution has not yet been studied. Starting from this assumption, we analyzed in this study only recovered patients, at two months after discharge, when theoretically, they should no longer have PH or altered RV function. However, we documented, in 7.69% of our patients, pathological values of esPAP and, in 10.28% of them, RVD, but it is to mention that we determined borderline values of these parameters in several other cases (higher than expected in this category of age), strongly correlated with the severity of initial lung damage [25]. The TTE estimated global prevalence of PH reported in the Rotherdam Study is 0.8% in patients aged between 65–75 years and of 2.6% in the general population [26], but we did not find data in younger patients.

Main responsible mechanisms for PH associated with COVID–19 are considered extensive pulmonary damage (due to interstitial and alveolar inflammation) as in PH of group 3 and alterations of pulmonary vasculature (induced by thrombotic/thromboembolic processes, endothelial injury, or, at least, hypoxic vasoconstriction), like in the PH of group 4, but also, in some cases, an additional factor, could be the consequences of positive end-expiratory pressure used in mechanical ventilation [5,6]. All our patients had interstitial and alveolar pulmonary injuries, but, when under 40% with TCT, COVID score between 2 and 10, and with O_2_ saturation over 87%, none of them needing mechanical ventilation. To limit the impact of thromboembolic processes, all our patients were treated, according to recommendations, with anticoagulants [27,28,29] both during hospitalization and 40 days after the discharge, but the endothelial dysfunction, microangiopathy, and microthrombosis could play an important role in the pathogenesis of PH. The contribution of the extensive inflammatory processes encountered in COVID-19 in the occurrence and perpetuation of PH has not yet been established, raising the hypothesis that several mechanisms responsible for PH of groups 3 and 4, associated with inflammation, are implicated in its pathogenesis. Our study suggests that elevated esPAP values and altered RV function could be encountered even after two months since the recovery from the acute phase of this infection. Surprisingly, the increased initial levels of inflammatory factors were strongly correlated with the elevation of esPAP and/or reduction of RV-GLS. The presence of these echocardiographic abnormalities, even after the recovery from mild Covid-19 pulmonary infections, raises the suspicion that inflammatory responses could be responsible for the persistence and further propagation of PH and RVD. It would be interesting what results regarding pulmonary hemodynamics and RV function could be obtained after a longer follow-up.

### Study Limitations

The main limit of our study is the small number of patients, but it was difficult to find more patients recovered from COVID-19, without associated pathology, and willing to take part in our investigation. Another limitation is that we do not have the baseline values of the TTE parameters because an accurate evaluation was not performed during hospitalization. As such, we do not have the exact values of esPAP before the infection. The third limitation is that, in this study, we relied only on the TTE assessment of esPAP and PVR and have not validated our data with invasive methods–right heart catheterism, but, due to the pandemic restrictions, we could not perform this procedure on a routine base. The last limitation is that we have not followed our patients for a longer time, but that remains to be accomplished in the future.

## 5. Conclusions

Pulmonary hypertension is a frequent complication of COVID-19, occurring even in cases with moderate pneumonia. Its evolution over time is not yet well established, but it seems to last longer than it has been appreciated. It appears to be related to the severity of the initial pulmonary injury and the extent of the inflammatory response.

## Figures and Tables

**Table 1:** Demographic and clinical characteristics of the study population.

Characteristics	All Patients	Patients with PH and Borderline esPAP	Patients with RVD and Borderline RVF
Number of cases	91	7 (7.69%) 18 (19.78%)	10 (10.28%) 12 (13.18%)
Gender	Male 44 (48.35%)	Male 4 + 10	Male 5 + 7
	Women 47 (51.64%)	Women 3 + 8	Women 4 + 6
Age (median)	46 (40–50)	46 (40–50)	48 (42.75–50.5)
BMI	26.8 (23.5–31.8)	27.8 (23.1–31.8)	29.6 (24.97–32.1)
TCT global score	4 (3–5)	6 (5–7)	6 (5–8)
˂5 points	58–63.73%	3–12%	1–4.54%
5–10 points	33–36.46%	22–88%	21–95.46%
Echocardiography Results			
RA diameter	3.5 (3.2–3.8)	3.8 (3.7–4.05)	3.8 (3.7–4.12)
RV diameter	2.8 (2.7–3)	3 4 (3.3–3.7)	3.4 (3.27–3.82)
TRVmax	2.3 (2.1–2.6)	2.71 (2.66–3.25)	2.72 (2.68–3.27)
esPAP	26.16 (22.64–30.24)	34.4 (33.41–47.38)	34.6 (33.78–47.77)
PVR	1.5 (1.2–2)	2.5 (2–3.3)	2.25 (2–3.2)
TAPSE RV-GLS	23 (22–24) −29 (−30–−28)	20 (17.75–22) −26 (−27–−17.5)	20 (17.37–21.25) −25 (−27–−17)
Laboratory Results			
Leukocites (/uL)		5820 (3740–8060)	6025 (4570–7477.5)
Lymphocites (/uL)	1790 (1430–2430)	1740 (1160–2250)	1790 (1407.5–2310)
D-dimer (ng/mL)	0.32 (0.29–0.4)	0.41 (0.34–0.42)	0.41 (0.33–0.42)
CRP (mg/L)	32.63 (10.78–42.73)	45.58 (33.59–48.83)	45.81 (42.64–49.16)
Fibrinogen (g/L)	2.92 (2.47–3.58)	3.78 (3.26–4.08)	3.88 (3.58–4.33)
Interleukin-6 (pg/mL)	5.6 (3.2–8.1)	8.9 (8.7–9.65)	9 (8.7–10.32)
O_2_ saturation (%)	96 (96–98)	93 (90.5–96)	93 (90–95.25)
Time to normal PCR	15 (14–17)	18 (17–19.5)	18 (17–21.75)

Legend: Body mass index—BMI. Thorax computer tomography—TCT. Right atrium—RA. Right ventricle—RV. Maximum tricuspid regurgitation velocity—TRVmax. Estimated systolic pressure in pulmonary artery—esPAP. Pulmonary vascular resistance—PVR. Tricuspid annular plane systolic excursion—TAPSE. Right ventricular global longitudinal strain—RV-GLS. C reactive protein—CRP.

**Table 2:** Relationship between esPAP and RV-GLS and the studied parameters.

Parameter	esPAP		RV-GLS	
	*r* (95%CI)	*p*	*r* (95%CI)	*p*
TCT Covid score	*r* = 0.608 (0.425; 0.751)	˂0.001	*r* = 0.547 (0.350; 0.699)	˂0.001
CRP	*r* = 0.629 (0.472; 0.767)	˂0.001	*r* = 0.546 (0.360; 0.702)	˂0.001
Interleukin-6	*r* = 0.748 (0.594; 0.865)	˂0.001	*r* = 0.524 (0.300; 0.708)	0.001
Fibrinogen	*r* = 0.428 (0.207; 0.610)	˂0.001	*r* = 0.483 (0.277; 0.664)	0.001
Days until negativation	*r* = 0.598 (0.444; 0.743)	˂0.001	*r* = 0.540 (0.348; 0.711)	0.001

Legend: estimated systolic pressure in pulmonary artery—esPAP. Right ventricular global longitudinal strain—RV-GLS. Thorax computer tomography—CT. C reactive protein—CRP. CI—confidence interval. *p*—statistical significance. *r*—correlation coefficient. Spearman’s rank correlation test.

**Table 3:** Multivariate linear regression analysis.

Variable	β	±SE	*p*	95%CI for β
Multivariate linear regression analysis of esPAP				
TCT COVID score	1.488	0.295	0.001	0.903; 2.074
D-Dimers	−12.569	5.444	0.023	−23.395; −1.744
Fibrinogen	−1.102	0.528	0.040	−2.151; −0.052
Interleukin-6	0.526	0.175	0.004	0.178; 0.875
O2 saturation	−0.323	0.133	0.017	−0.587; −0.058
PVR	7.046	0.774	0.001	5.506; 8.586
Multivariate linear regression analysis of RV-GLS				
TCTCOVID score	0.556	0.138	0.001	0.281; 0.830
Interleukin-6	−0.161	0.066	0.017	−0.292; −0.030
TRVmax	−16.724	3.221	0.001	−23.129; −10.318
esPAP	1.138	0.180	0.001	0.780; 1.496
RA diameter	−1.582	0.446	0.001	−2.469; −0.694
TAPSE	−0.361	0.101	0.001	−2.469; −0.694

Legend: CI—confidence interval. β—regression coefficient. SE—standard error. *p*—statistical signification. Estimated systolic pressure in pulmonary artery—esPAP. Thorax computer tomography—TCT. Pulmonary vascular resistance—PVR. Real-time reverse transcriptase–polymerase chain reaction—PCR. Right ventricular global longitudinal strain—RV-GLS. Tricuspid annular plane systolic excursion—TAPSE. Statistical method: multivariate stepwise linear regression (Akaike information criteria).

## Data Availability

Data are available under https://www.preventim.ro/public/.
